# Spontaneous Visual Acuity Recovery After Cilioretinal Artery Occlusion Combined With Central Retinal Vein Occlusion in a Young Patient

**DOI:** 10.7759/cureus.23476

**Published:** 2022-03-25

**Authors:** Myron Z Markakis, Georgios Bontzos, Michail Nodarakis, Dimitrios Dimopoulos, Andreas Zacharioudakis, Pavlos Koutentakis

**Affiliations:** 1 Department of Ophthalmology, Venizeleio General Hospital of Heraklion, Heraklion, GRC

**Keywords:** spontaneous recovery, fundus fluorescein angiography, visual acuity, central retinal vein occlusion, cilioretinal artery obstruction

## Abstract

This work illustrates the case of cilioretinal artery occlusion (CilRAO) combined with central retinal vein occlusion (CRVO) in a young patient that resolved spontaneously. A 17-year-old male with an unremarkable medical history presented with acute painless loss of vision unilaterally. Upon ophthalmologic examination, retinal hemorrhages in all four quadrants and edema extending from the optic disc to the macula were reported. Using optical coherence technology (OCT) imaging and fundus fluorescein angiography (FFA), combined CilRAO/CRVO was diagnosed. The full medical evaluation was unremarkable. Within the next month, the patient had regained full visual acuity (VA) in the affected eye, and the retinal findings resolved without intervention. Combined CilRAO/CRVO is a common vascular pathology in young, otherwise healthy patients. It is commonly considered a hemodynamic block in the capillary bed, hence its hopeful prognosis. Nonetheless, several risk factors have been proposed that need to be eliminated. Despite the initial alarming symptoms, young patients with CilRAO/CRVO should be monitored closely, and intervention should be resorted to when necessary.

## Introduction

The cilioretinal artery - originally described by Mueller in 1856 [1] - is a congenital vascular variant approximately present in one-third of normal eyes. It originates from either the short posterior ciliary arteries or the peripapillary choroid network.

Cilioretinal artery occlusion (CilRAO) is an uncommon variant of artery occlusion. It accounts for 5% of all retinal artery occlusions [2]. It is commonly divided into three groups: i) isolated CilRAO, ii) CilRAO associated with retinal vein occlusions, and iii) CilRAO associated with giant cell arteritis. When seen in younger patients, retinal arterial occlusions are associated with hypercoagulability, hemoglobinopathies, and ocular trauma [3].

While the exact mechanism of CilRAO remains unclear, there is a consensus that CilRAO should be interpreted as a hemodynamic block because of increased intraluminal pressure in the capillary bed [4]. There are no established guidelines regarding these cases, and management is mostly focused on targeting underlying causes.

This work illustrates a case of combined CilRAO/central retinal vein occlusion (CRVO) that resolved spontaneously with complete restoration of visual acuity (VA) in a healthy young patient.

## Case presentation

A 17-year-old male patient presented to the emergency eye clinic complaining of acute painless loss of vision in his right eye for two hours. He had no further systemic complaints, his personal and family history was unremarkable, and he was not under any medication. He mentioned similar symptoms earlier that month that resolved spontaneously within an hour. The patient did not seek medical consultation at that time. The patient had no comorbidities or history of systemic disorders and denied any previous or current use of isotretinoin.

The patient underwent a full ophthalmologic examination. His best-corrected visual acuity (BCVA) was 20/400 in the right eye and 20/20 in the left eye, and his intraocular pressure (IOP) was 16 mmHg in both eyes. Eye movement was not restricted, and no pain was elicited. Pupillary light reflexes were normal.

**Figure 1:**
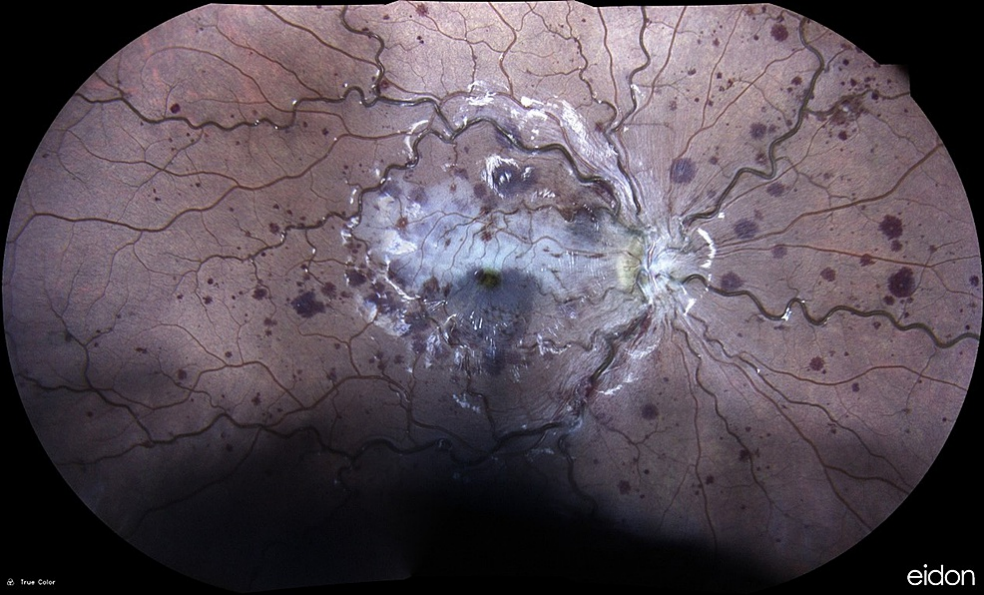
Patient's right fundus upon presentation Diffuse retinal hemorrhages, vein tortuosity, and retinal edema of the macular area in the distribution of cilioretinal artery perfusion.

**Figure 2:**
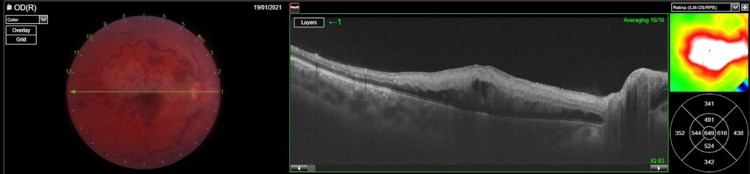
Optical coherence tomography of the patient's right eye during the acute phase of the occlusion We can observe subretinal fluid along with thickening of the outer retinal layers compatible with macular ischemia. The inner retina also appears disrupted due to macular edema.

Fundoscopy in his right eye revealed diffuse retinal edema extending from the macular area to the optic disc corresponding to a pale-yellow area on the course of a cilioretinal artery. Retinal veins were dilated and tortuous, and flame hemorrhages were present in all four quadrants. Fundus examination showed optic disc edema and increased tortuosity of the retinal veins and a few retinal hemorrhages (Figure 1). No rescue therapy including ocular massage or IOP-lowering medications was indicated at this time. Optical coherence technology (OCT) imaging was also obtained, where diffuse retinal thickening was observed, mostly in the outer retinal layers (Figure 2). Combined central retinal vein occlusion and cilioretinal artery occlusion was suspected.

Visual field testing using Dicon perimetry revealed extensive ceco-central depression. Slit-lamp examination showed no abnormal findings in both eyes.

**Figure 3:**
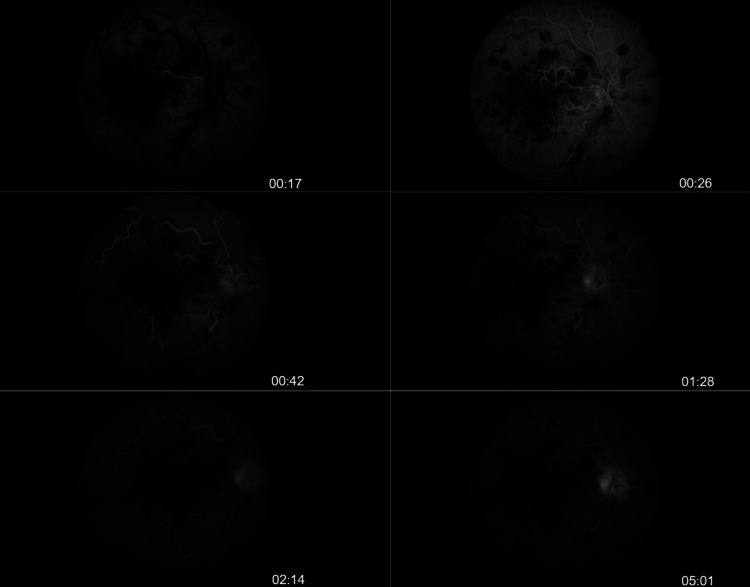
Patient's fundus fluorescein angiography Delayed capillary perfusion during early phases and delayed vein filling are shown. We can also observe that the area of cilioretinal perfusion remains hypofluorescent, as a result of macular thickening and ischemia. We observe that despite the presence of dye within the cilioretinal artery, there is delayed filling and marked defects in its distribution.

Fundus fluorescein angiography (FFA) was performed on the next day to validate the diagnosis. Delayed filling of retinal veins, indicative of nonischemic CRVO, was marked. Flow within the cilioretinal artery was present, while the surrounding area was ischemic. No optic disc leakage or other signs of inflammation were observed (Figure 3).

**Figure 4:**
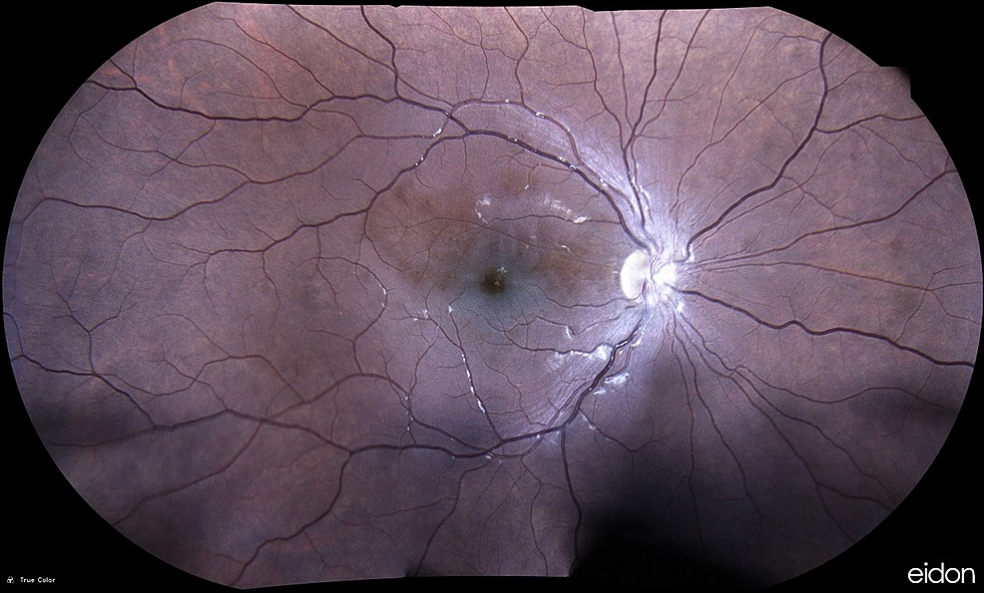
Right fundus photo two months after the event Fundus findings and hemorrhages were mostly diminished.

The patient underwent a full medical evaluation to determine the possible causes of retinal vessel obstruction. Standard laboratory tests to investigate systemic causes including tuberculosis, syphilis, cytomegalo­virus (CMV), and HIV were undertaken, and all were negative. Further workup included full blood tests, complete cardiologic evaluation (including electrocardiogram, 24-hour Holter device, and heart echo), thrombophilia screening, inflammatory disease control (including autoantibodies, homocysteine levels, angiotensin-converting enzyme levels, and infectious screening for cat scratch disease), and tuberculosis and HSV-VZV testing, which were also negative. No intervention of any form was indicated at that time. Thereafter, the patient was followed up on a regular outpatient basis. His VA in the right eye showed a marked improvement reaching 20/32 after one week. One month later, his VA rose to 20/20 in the right eye, but the ceco-central depression persisted in visual field testing. At follow-up, two months after onset, the scotoma was almost diminished, as did the retinal findings including the hemorrhages (Figure 4). His condition remained stable on his last examination, nine months after the initial episode, and FA showed normal capillary filling without signs of occlusion or inflammation.

## Discussion

In the present study, we presented a case of combined CilRAO/CRVO where remarkable fundus findings and significant visual loss, probably due to CMO, were spontaneously recovered. The initial findings in our case were similar to those in CRVO with an additional retinal infarct in the distribution of the occluded cilioretinal artery.

Visual acuity reduction upon presentation might be attributed mostly to the macular edema caused by the CRVO. It has been reported that eyes with nonischemic CRVO without foveal involvement by CilRAO have marked visual improvement over the follow-up period compared with eyes with retinal infarcts in the foveal area. The latter usually results in permanent central scotoma [4]. It has also been shown that ceco-central scotoma is the most common defect associated with CilRAO [4]. To further understand why some eyes with combined CilRAO/CRVO develop permanent visual loss, we have to consider the retinal tolerance to acute ischemia. It has been found that ischemia lasting for less than 100 minutes can cause reversible retinal damage that can recover with full retinal function [5]. After that point, any further delay in reestablishing the blood supply can cause permanent damage.

As mentioned before, three forms of CilRAO have been described: the embolic form caused by carotid atheromatosis and two combined forms that are associated either with CRVO or anterior ischemic optic neuropathy (AION) [2,3]. In the case of combined CilRAO/CRVO, the exact pathogenesis is controversial. It has been suggested that CilRAO is the aftermath of the raised capillary pressure caused by CRVO and the increase in intraluminal pressure of the capillary bed. Hence, CilRAO is a functional block of hemodynamic nature [4]. Another hypothesis assumes that the decreased retinal circulation, after the initial drop in perfusion pressure of the cilioretinal artery in the event of CilRAO, i.e., vasospasm, results in venous stasis and thrombosis [4]. In the present case, signs of CRVO were notable from the beginning, with increased retinal tortuosity and retinal hemorrhages, which persisted after the arteritic disease resolution, favoring the functional block hypothesis.

It is essential to mention that CilRA is derived from the choroidal circulation, arising directly from the ophthalmic artery, and unlike the central retinal artery, it does not have protective mechanisms against blood flow fluctuations. Moreover, there is no vortex vein obstruction. Therefore, the lack of compensatory mechanisms of CilRA to sudden perfusion pressure variations makes it susceptible to hypotensive events or sudden variations of perfusion pressure [4]. Finally, another fact that requires attention is that the trajectories of the large retinal arteries tend to share routes with those of the axon bundles in the nerve fiber layer. The retinal architecture makes, however, the branch arteries particularly vulnerable, since their peripheral borders are crossed by a significant number of ganglion cell axons [5].

Various risk factors have been proposed for cases of combined CilRAO/CRVO. Atherosclerosis plays an important role in older individuals. Thrombophilia, vasculitis, and autoimmune diseases are also considered. In younger patients, homocysteine levels, infectious diseases such as syphilis, patent foramen ovale, Flammer and HELLP syndromes, and other rare diseases should be considered [4]. Nevertheless, otherwise healthy individuals - usually younger persons - may also present with combined CilRAO/CRVO [6].

The management of combined CilRAO/CRVO remains debatable. Regarding arterial occlusion, the cornerstone is to restore blood supply. To that end, intravenous anticoagulation treatment (heparin and low-molecular-weight heparin) has been used, often together with isovolumic hemodilution when venous occlusion was also present [1]. Brazitikos et al. selectively administered antiplatelet agents in some patients [6]. At the same time, it is crucial to address the causative agent of the occlusion with targeted treatment (antihypertensives, systemic steroids, etc.) [6]. Hyperbaric oxygen therapy is another successful alternative in cases with vision loss [7]. In the present study, no specific risk factors were identified, while the CilRAO was transient, and the venous signs along with the drop in VA improved early, so we decided to carefully monitor this patient without any intervention.

The prognosis in combined CilRAO/CRVO is variable. As discussed above, patients with ischemic CRVO show less visual improvement than patients with nonischemic type [4]. In addition, the duration and severity of retinal ischemia from the CilRAO is a decisive factor. In this patient, flow within the CilRA reestablished soon after the initial symptom as the FFA verified [5]. It remains to be elucidated whether the condition will remain stable or further workup is required. Evaluation of these cases should include careful workup to identify the potential causes of the obstruction and examination of the unique association between the CilRA and central retinal vein to determine prognosis and functional outcomes.

## Conclusions

Visual acuity recovery in cases of combined CilRAO/CRVO mainly depends on the duration of the retinal ischemia caused by the CilRAO and the macular edema caused by the CRVO. The pathogenesis of this combined entity is not yet fully understood, and further studies should be undertaken. As in most cases of vasculopathy, various systemic risk factors have been suggested that should be identified. Management remains controversial, and it revolves around restoring blood supply and addressing the underlying pathologies.
